# Hepatic Epithelioid Hemangioendothelioma Mimicking Metastatic Carcinoma on Liver Biopsy: A Case Report

**DOI:** 10.7759/cureus.106125

**Published:** 2026-03-30

**Authors:** Saikat Mitra, Ashish Kawthalkar, Shantanu Pande

**Affiliations:** 1 Pathology, All India Institute of Medical Sciences, Nagpur, IND; 2 Nuclear Medicine and Molecular Imaging, All India Institute of Medical Sciences, Nagpur, IND

**Keywords:** diagnostic pitfall, hepatic epithelioid hemangioendothelioma, immunohistochemistry, liver biopsy, metastatic carcinoma, vascular tumor

## Abstract

Hepatic epithelioid hemangioendothelioma (HEHE) is a rare malignant vascular tumor that frequently poses a diagnostic challenge due to its close resemblance to metastatic carcinoma, particularly on limited biopsy material.

We report the case of a 42-year-old woman who presented with constitutional symptoms and gastrointestinal bleeding. Imaging revealed multiple bilateral hepatic lesions, clinically interpreted as metastases from an unknown primary. The liver biopsy revealed cords and tubular structures composed of epithelioid cells within a reactive hepatic background. Immunohistochemistry revealed isolated CK7 positivity, leading to an initial impression of metastatic carcinoma or intrahepatic cholangiocarcinoma. Extensive clinic-radiological work-up failed to identify a primary malignancy. Review of histology highlighted blister cells and intracytoplasmic vacuoles, and subsequent endothelial marker positivity confirmed the diagnosis of HEHE.

This case underscores the importance of recognizing histologic clues and the diagnostic pitfall in HEHE, which can lead to misdiagnosis and inappropriate management.

## Introduction

Hepatic epithelioid hemangioendothelioma (HEHE) is a rare malignant endothelial neoplasm with a prevalence of less than one per million [1]. It is characterized by an intermediate biological behavior between hemangioma and angiosarcoma. HEHE demonstrates a variable clinical course ranging from indolent behavior to progressive disease with metastasis. More than two-thirds of cases present as multifocal hepatic lesions involving both lobes. Radiologically, it commonly presents as multiple peripheral nodules with capsular retraction, frequently leading to an initial diagnosis of metastatic carcinoma. Histologically, the epithelioid morphology, cord-like growth pattern, and presence of intracytoplasmic lumina closely mimic adenocarcinoma. Diagnostic difficulty is compounded by aberrant cytokeratin expression, including CK7 positivity in a subset of tumors. Awareness of these pitfalls is essential, especially when evaluating small core biopsies. Molecularly, the characteristic *WWTR1::CAMTA1* gene fusion has been identified in the majority of cases and supports its distinct pathogenesis [2].

We report a case of HEHE initially misdiagnosed as metastatic carcinoma, highlighting the critical histologic and immunohistochemical (IHC) clues required for accurate diagnosis.

## Case presentation

A 42-year-old woman presented with a history of fever and gastrointestinal bleeding, for which she received symptomatic treatment. Although her acute symptoms subsided, she continued to experience persistent generalized weakness. There was no history of abdominal pain or jaundice. An ultrasonography performed as part of a routine imaging investigation at an outside hospital revealed multiple hypoechoic lesions involving both hepatic lobes, suggestive of metastatic disease. The imaging films were unavailable for review. Serum tumor markers, including alpha-fetoprotein (AFP) (1.3 ng/mL, reference: <10 ng/mL), CA 19.9 (3.86 U/mL, reference: <37 U/mL), CEA (1.24 ng/mL, reference: <3 ng/mL), CA-125 (14 U/mL, reference: <35 U/mL), were tested, but all of them were within normal limits. In view of a suspected carcinoma of unknown primary with liver metastasis, a CT-guided biopsy was performed from one of the liver lesions. Histopathological examination performed initially at an outside laboratory suggested a possibility of malignant epithelial neoplasm as the liver tissue core was infiltrated by epithelioid cells arranged in cords and vague tubular structures. Subsequently, a panel of IHC markers was performed to further characterize the tumor. The IHC evaluation demonstrated positivity for CK7 in the tumor cells. CK20, CDX-2, GCDFP-15, GATA3, TTF-1, Napsin-A, WT-1, and PAX8 were negative, which made metastasis from lower gastrointestinal tract adenocarcinoma, breast carcinoma, lung adenocarcinoma, and genitourinary adenocarcinoma less likely. CK19 and Glypican-3 negativity made the possibility of cholangiocarcinoma or multifocal hepatocellular carcinoma less likely. Based on morphology and CK7 positivity, the possibility of metastatic adenocarcinoma from the upper gastrointestinal tract was considered from the outside histopathology laboratory.

**Figure 1:**
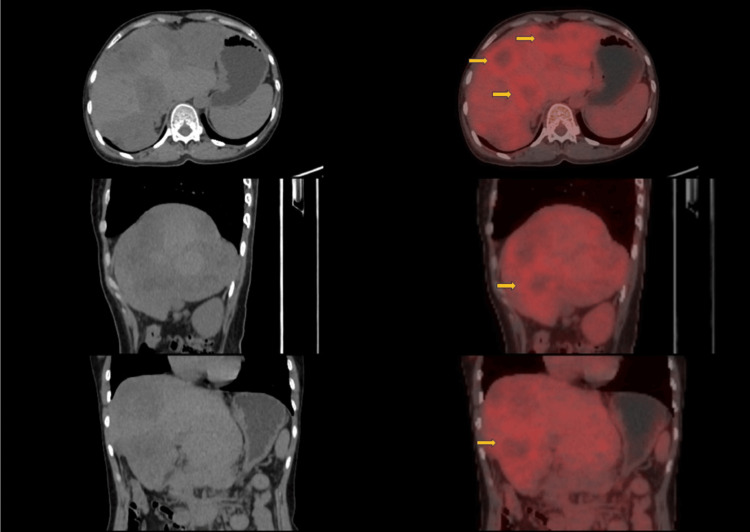
18F-FDG PET-CT image of the abdomen The PET image shows the axial (top row) and coronal (middle and bottom rows) sections. The left panel demonstrates contrast-enhanced CT images, while the corresponding right panel shows fused PET-CT images. There are multiple, heterogeneously enhancing hepatic lesions involving both lobes, predominantly the right lobe of the liver, with intense and heterogeneous FDG uptake within the lesion (yellow arrows), consistent with a metabolically active tumor. Background liver parenchyma shows comparatively lower physiological uptake. FDG: fluorodeoxyglucose, PET-CT: positron emission tomography-computed tomography.

Further evaluation with PET-CT revealed multiple low-grade fluorodeoxyglucose (FDG)-avid lesions confined to the liver, with no evidence of extrahepatic disease (Figure 1).

Upper gastrointestinal endoscopy and mammography did not reveal any abnormalities. Despite the absence of a demonstrable primary tumor, chemotherapy was advised; however, the patient defaulted due to financial constraints. Later, she was referred to our center for further evaluation and treatment.

**Figure 2:**
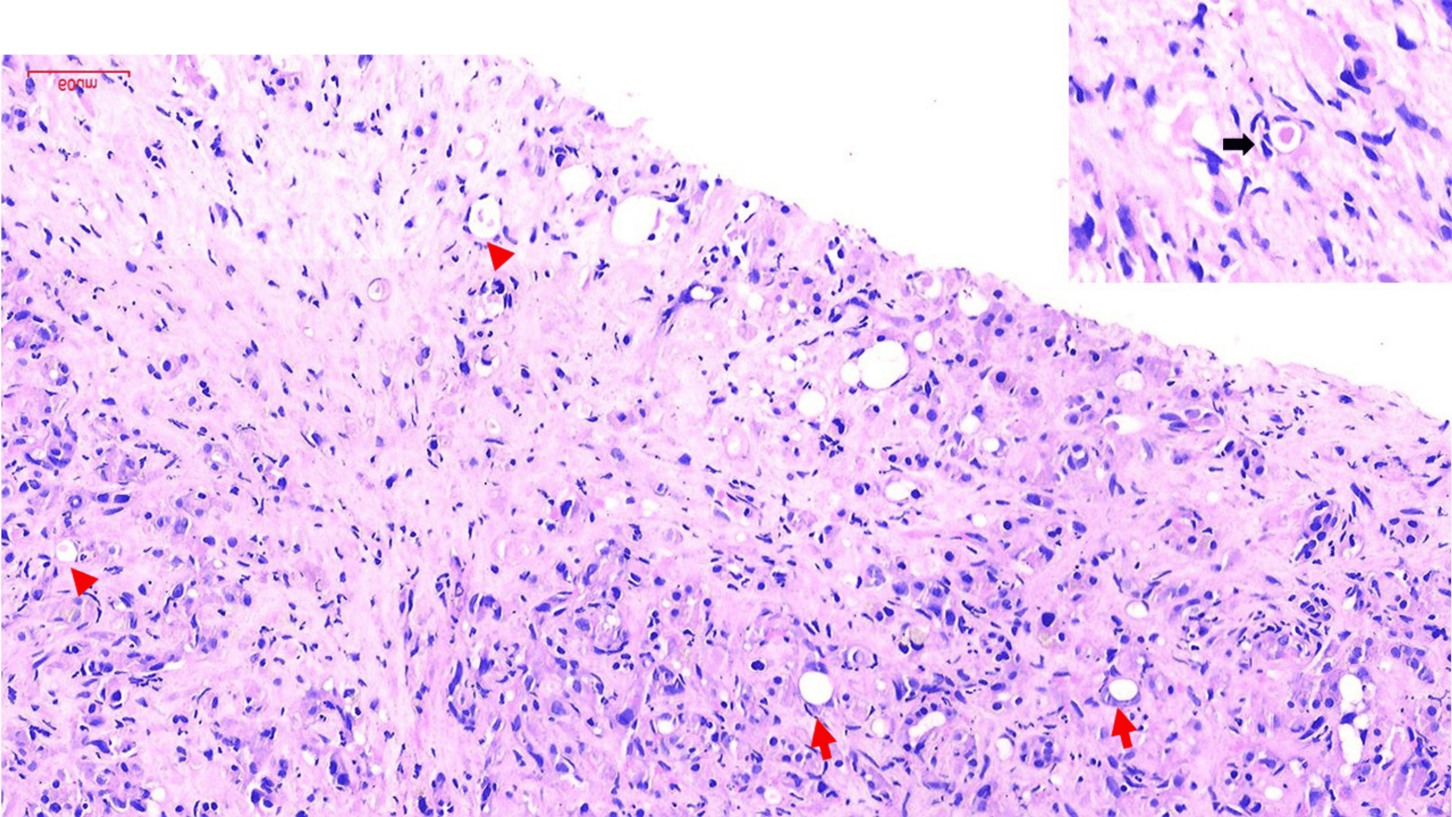
Photomicrograph of the hepatic lesion (H&E, 100x) The liver biopsy shows singly scattered and single cords of tumor cells over a hyalinized stroma. The tumor cells show cytoplasmic lumina with moderate nuclear atypia (red arrow). A few scattered tumor cells show red blood cells in the cytoplasmic lumina (arrowhead). The tumor cells with cytoplasmic lumina and red blood cells are highlighted in the inset (black arrow).

In view of the discordance between histopathological findings and clinical-radiological work-up, the liver biopsy was reviewed. Careful re-evaluation revealed distorted hepatic lobular architecture, without any well-defined lesion. There was florid ductular proliferation and hepatocyte loss with foci of macrovesicular steatosis. However, the tumor was seen to be composed of cords of epithelioid cells and singly scattered tumor cells within a hyalinized stroma. Histopathological findings on H&E stain are shown in Figure 2.

**Figure 3:**
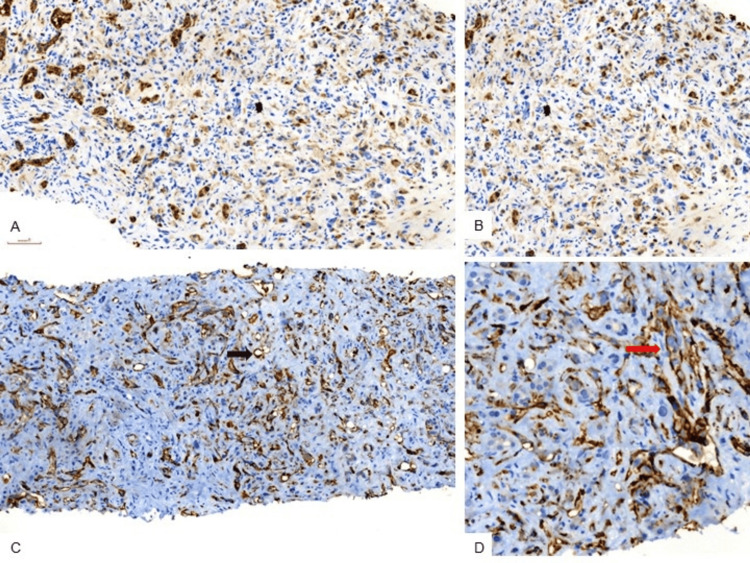
Immunohistochemistry for CK7, CD34, and CD31 (A; IHC 40x) and (B; IHC, 100x): CK7 immunohistochemistry highlights reactive bile ducts (strong cytoplasmic positivity), while tumor cells show weak to focal staining. (C; IHC, 40x): CD34 immunohistochemistry demonstrates diffuse membranous positivity in tumor cells arranged in cords and singly scattered cells, confirming endothelial differentiation. The singly scattered tumor cells are labelled with a black arrow. (D; IHC, 200x): CD31 immunohistochemistry demonstrates diffuse membranous positivity in tumor cells arranged in cords and singly scattered cells. The cords of the tumor cells are labelled with a red arrow.

Several of the tumor cells showed intracytoplasmic lumina, containing fragmented red blood cells. The tumor cells showed moderate nuclear anaplasia; however, mitotic activity was rare, and tumor necrosis was absent. CK7 immunohistochemistry was repeated and demonstrated strong positivity in the reactive and proliferating benign bile ducts, while the tumor cells were negative to focally weakly positive. Subsequent immunostaining for endothelial markers demonstrated diffuse membranous positivity for CD31 and CD34. IHC findings are depicted in Figure 3. 

A final diagnosis of HEHE was rendered. Since the patient had multifocal hepatic lesions without any extrahepatic metastasis, a watchful follow-up was advised, and no systemic therapy was advised. The patient is currently under observation. IHC for CAMTA1 could not be done due to the unavailability of the primary antibody. A molecular study could not be performed due to financial constraints.

## Discussion

HEHE is a well-recognized diagnostic mimicker of carcinoma, particularly in small liver biopsies [2]. Weiss and Enzinger first described HEHE as a vascular tumor often mistaken for carcinoma [3]. The authors performed ultrastructural evaluation and confirmed the endothelial nature of the tumor by demonstrating cells surrounded by basal lamina, dotted with surface pinocytotic vesicles, and Weibel-Palade bodies. Despite increased awareness and advances in immunohistochemistry, misdiagnosis remains common.

HEHE presents with an incidentally detected multifocal hepatic mass, with a clear female predominance, and affects middle-aged individuals. A series of eight cases of HEHE from India showed a mean age of 49 years, with a female-to-male ratio of 7:1, and multifocal hepatic lesions were seen in two-thirds of the patients [4]. Several series have shown that the most common clinical differential diagnoses include metastatic carcinoma, intrahepatic cholangiocarcinoma, or multifocal hepatocellular carcinoma [5]. In the present case, exclusive liver involvement with no identifiable primary further complicated interpretation.

Histologically, HEHE exhibits epithelioid cells arranged in cords, nests, and short strands within a myxohyaline stroma [6]. Intracytoplasmic lumina, representing primitive vascular channels, are a critical diagnostic clue but may be subtle or overlooked, particularly when masked by reactive hepatocytes and ductular proliferation, as seen in our case. These intracytoplasmic lumina can sometimes contain fragmented RBCs. These cells are referred to as "blister cells." Macrovesicular steatosis displaces hepatocyte nuclei to the periphery and may mimic the intracytoplasmic lumina of HEHE, leading to misinterpretation as steatosis.

Immunohistochemistry for CK7 can act as a double-edged sword and can lead to a diagnostic dilemma. Aberrant CK7 expression in malignant vascular tumors is increasingly recognized. Lee et al. demonstrated that both pan-cytokeratin (AE1/AE3) and CK7 positivity are common in primary hepatic malignant vascular tumors. CK7 expression was noted in 56% of HEHE cases in their cohort [7]. In our case, isolated CK7 positivity resulted in extensive evaluation for metastatic carcinoma and delayed diagnosis. Moreover, our case was complicated by the exuberant proliferation of the bile ducts in the form of small tubules, which were also strongly stained with CK7, and were probably mistaken for metastatic carcinoma. Endothelial markers, such as CD31, CD34, and ERG, remain the most reliable IHC tools for diagnosis. *WWTR1::CAMTA1* gene fusion, resulting from a t(1;3)(p36;q25) translocation, is a characteristic feature of epithelioid hemangioendothelioma, found in as many as 90% of cases [8]. Rare hepatic tumors harbor a *YAP1::TFE3* fusion. CAMTA1 immunostaining or molecular confirmation of *WWTR1::CAMTA1* fusion provides additional specificity when available. However, in resource-limited settings, careful morphologic assessment combined with a limited but targeted immunopanel is often sufficient. The present case reinforces recommendations from expert consensus statements, including the ESMO consensus on epithelioid hemangioendothelioma, which emphasize early consideration of HEHE in multifocal liver tumors and routine use of endothelial markers when epithelial markers yield inconclusive results [9].

Management of HEHE remains challenging due to its rarity, unpredictable biological behavior, and lack of standardized treatment guidelines. Contemporary literature emphasizes an individualized, multidisciplinary approach. Surgical resection remains the preferred treatment for patients with localized or unifocal disease and is associated with favorable long-term outcomes. However, the majority of patients present with multifocal hepatic involvement, limiting resectability. In such cases, liver transplantation has emerged as an effective therapeutic option, even in the presence of limited extrahepatic disease, with reported long-term survival rates comparable to or superior to those achieved with resection [10,11]. For patients with indolent, asymptomatic disease, a conservative “watchful waiting” strategy has been advocated. Systemic therapies, including conventional chemotherapy, have shown limited and inconsistent benefit [10,11]. More recent approaches have focused on anti-angiogenic agents and targeted therapies, though evidence remains largely anecdotal or derived from small series. 

The present case highlights an important diagnostic lesson: in the setting of multifocal liver lesions without an identifiable primary tumor, routine inclusion of endothelial markers in the immunopanel should be considered early, even when epithelial markers show focal positivity.

## Conclusions

HEHE is a rare vascular tumor that poses a significant diagnostic challenge on liver biopsy. Aberrant keratin positivity represents a major pitfall, frequently leading to misdiagnosis as metastatic carcinoma or cholangiocarcinoma. Recognition of subtle histologic features, such as intracytoplasmic lumina, luminal RBCs, and early incorporation of endothelial markers, is essential for accurate diagnosis. Increased awareness of this entity can prevent inappropriate treatment and improve patient management.
